# Evaluation of Cement Composites with Heavy Metal-Contaminated Recycled Aggregate: Toward Sustainable Utilization

**DOI:** 10.3390/ma18245533

**Published:** 2025-12-09

**Authors:** Tilen Turk, Petra Štukovnik, Marjan Marinšek, Violeta Bokan Bosiljkov

**Affiliations:** 1 Faculty of Civil and Geodetic Engineering, University of Ljubljana, Jamova 2, 1000 Ljubljana, Slovenia; tilen.turk@fgg.uni-lj.si (T.T.); petra.stukovnik@fgg.uni-lj.si (P.Š.); 2 Faculty of Chemistry and Chemical Technology, University of Ljubljana, Večna pot 113, 1000 Ljubljana, Slovenia; marjan.marinsek@fkkt.uni-lj.si

**Keywords:** recycled aggregate, concrete, heavy metals, tailings, setting time

## Abstract

The use of recycled aggregate provides clear environmental advantages but may introduce chemical interactions that influence cement hydration, particularly when the material originates from mining by-products containing heavy metals. This study examines cementitious composites containing different volume fractions of recycled aggregate derived from Pb–Zn mine tailings and identifies the mechanisms responsible for the observed early-age hydration delay. The recycled aggregate was characterized using XRD, hydration was monitored through ultrasonic pulse velocity (UPV) and temperature evolution, mechanical performance was assessed at 1, 3, and 7 days, and phase evolution was interpreted using SEM-EDS and thermodynamic equilibrium modeling (GEMS/Cemdata18). The results show that heavy-metal-bearing phases (Zn-, Pb-, and Fe-sulfides/sulfates) promote the formation of metastable metal–silicate complexes, temporarily lowering the oxidation potential and delaying setting by up to 28 h in mixtures containing 100% recycled aggregate. Early-age strength was substantially reduced; however, by day 7, all mixtures except that with 100% recycled aggregate approached the strength of the reference mixtures with natural aggregate. Despite these effects, recycled aggregate can be safely incorporated at replacement levels up to 25 vol.%, which preserves acceptable fresh and hardened properties. Nevertheless, the presence of persistent sulfate-bearing phases (e.g., epsomite, anglesite) indicates a potential for long-term sulfate release and associated durability risks, warranting further investigation.

## 1. Introduction

Concrete is the world’s most widely used construction material; therefore, its production from primary sources—namely cement clinker, water, and natural aggregates—has a significant impact on the environment and the conservation of natural resources [1]. Moreover, several European directives encourage the construction sector to reduce its carbon footprint, promoting the use of secondary or recycled materials in new structures [2]. Recycled materials are now increasingly utilized in cement production. In addition, to further lower the carbon footprint of concrete, the use of recycled aggregate has gained attention as a promising approach in the development of carbon-neutral concrete [3].

Recycled aggregates can originate from various industrial processes [4,5,6]. However, the most commonly used recycled aggregate for concrete production is obtained from the demolition or renovation of structures with a chemical composition similar to that of conventional concrete [3]. Studies using such recycled aggregates have shown that their use may present certain limitations and affect concrete quality [7,8,9]. Recycled concrete aggregates typically have a rough surface texture and high porosity. At higher replacement ratios, this increased porosity can lead to durability issues in the resulting concrete [7]. Nevertheless, when high-quality recycled aggregates derived from high-strength concrete are used, the compressive strength of the new concrete remains largely unaffected. Therefore, to achieve the desired concrete properties, the construction and demolition waste used as aggregate should be thoroughly characterized [8,9].

Recycled aggregate can also originate from uneconomical by-products of mining operations, commonly known as tailings. Tailings typically exhibit highly heterogeneous chemical compositions that can vary substantially between mining sites [10]. The extraction of metallic minerals depends strongly on the type of metal being recovered and generally involves a combination of oxidative and reductive processes. Moreover, ores contain multiple mineral phases that can interact during processing, making metal extraction inherently inefficient. For example, the extraction of ores rich in sphalerite and galena begins with grinding, followed by flotation to separate galena- and sphalerite-rich fractions [11]. In the first stage of flotation, various surfactants are added to modify the surface charge of mineral particles, enabling the sedimentation of other ores such as pyrite, smithsonite, and wulfenite. In the subsequent stage, depressants are introduced to selectively separate sphalerite from galena. The sedimented fraction of the ore, being economically unattractive, is typically discarded as tailings. This fraction contains different carbonates and residual metals present in the original ore. In the presence of oxygen and at elevated temperatures, however, the galena-rich portion undergoes oxidation. The resulting lead oxide is then reduced in coke-fired furnaces to produce elemental lead [12]. Consequently, tailings may contain various impurities, including ore-bound heavy metals and chemical residues from extraction processes [13,14]. When such materials are used as recycled aggregates in concrete production, these impurities can interact with cement hydration products, leading to altered properties of both fresh and hardened concrete. Recent studies investigating the incorporation of recycled materials derived from mining industries into concrete have demonstrated that heavy metals present in raw materials can prolong setting time and significantly affect the early-age strength of concrete [15,16,17,18,19,20,21].

Such a phenomenon could occur due to change in oxidation potential. Oxidation potential is an electrochemical parameter describing the tendency of the chemical environment to donate or accept electrons. In cementitious systems, oxidation potential is strongly dependent on pH; even slight fluctuations can shift redox equilibria and consequently influence redox-sensitive species present in the pore solution [22]. Redox-active species such as Fe^2+^ or Fe^3+^ can, depending on the oxidative conditions of the pore solution, alter the nucleation processes of C–S–H by adsorbing onto growing hydrates, incorporating between layers, or directly interacting with their structure [23], while the general mechanisms of C–S–H nucleation and ion sorption are well-described in Refs. [24,25]. Moreover, reduction of sulfate species can cause delayed ettringite formation and alter the hydration of C_3_A.

Studies investigating the use of recycled aggregates derived from mining sites have also reported the immobilization of heavy metals within the C–S–H gel. This phenomenon not only contributes to more sustainable concrete production through improved natural resource management but also provides a means of safer heavy metal waste stabilization [26,27]. Consequently, research has also focused on assessing the durability of such concretes, particularly under frost action and carbonation exposure. These studies confirmed that the leaching of heavy metals is directly proportional to their concentration within the aggregate when the concrete matrix undergoes degradation [28,29,30].

The use of recycled aggregates originating from ore mining is expected to increase over the coming decade. However, despite increasing interest, significant knowledge gaps remain. Existing studies have primarily focused on mechanical performance or environmental aspects, while early hydration mechanisms, particularly the formation of metastable heavy-metal–silicate complexes, sulfate-induced retardation, and the ionic evolution of pore solution species, are still poorly understood. These processes are critical because they govern the setting behavior and early-age performance of cementitious composites incorporating mining-derived recycled aggregate.

Therefore, the present study aims to address this knowledge gap by: (1) characterizing the mineralogical and chemical composition of a recycled aggregate originating from Pb–Zn mine tailings, (2) assessing its influence on flowability, setting time, and early mechanical performance, (3) applying advanced analytical techniques (UPV, temperature evolution, XRD, SEM-EDS), and (4) employing thermodynamic modeling to elucidate the transient mechanisms associated with redox-active heavy-metal species during early hydration.

This integrated approach provides new insight into the early hydration behavior of cementitious composites containing heavy-metal-bearing recycled aggregates and identifies the optimal replacement level that does not adversely affect setting or strength development.

## 2. Materials and Methods

### 2.1. Aggregate Characterization

Two different aggregates were selected: a recycled aggregate (J) and a reference aggregate (R), both with a particle size fraction of 0/4 mm. The reference aggregate was chosen based on the mineralogical composition of the recycled aggregate and consisted of 99.9% dolomite and 0.1% calcite.

The selected aggregates were tested for the presence of organic matter in accordance with EN 1744-1 [31], using a 3% NaOH solution. A sieve analysis was performed on both aggregates according to EN 933-1 [32] and their particle density and water absorption were determined in accordance with EN 1097-6 [33]. The pH of the aggregates was measured following ASTM D6739-20 [34]. In addition, X-ray diffraction (XRD) analysis was conducted on the recycled aggregate (J), applied to its particle-size subfractions, to determine its mineralogical composition.

### 2.2. Mixtures Preparation and Fresh Properties

Ordinary Portland cement (CEM I according to EN 197-1 [35], produced by Alpacem, Trbovlje, Slovenia) was used as the binder. Its chemical composition was determined by X-ray diffraction (XRD) analysis. The cement paste–to–aggregate ratio was 40:60 vol.%, and the water-to-cement ratio (w/c) was 0.45. The aggregates were first oven-dried to constant mass and then pre-wetted to a near-SSD condition. Their moisture content was measured, and the mixing water was adjusted accordingly to maintain the prescribed effective w/c ratio of 0.45. The aggregate conditioning procedure followed the COST TU1404 RRT protocol [36]. A polycarboxylate-ether (PCE)–based superplasticizer (SP; TKK Cementol Expert Hiperplast 182, produced by TKK Slovenia, Srpenica, Slovenia) was added to achieve a flowability of 130 ± 5 mm (EN 1015-3 [37]) for the reference mixture containing aggregate R. The reference aggregate was partially replaced by 25 vol.%, 50 vol.%, 75 vol.%, and 100 vol.% of recycled aggregate. The SP dosage was kept identical to that of the reference mixture, except for mixture J (100% recycled aggregate), where the original dosage caused visible instability (segregation and bleeding). Therefore, the SP content was reduced to ensure a stable mixture. This adjustment limited PCE-induced retardation while maintaining the target w/c ratio.

The mixing procedure and the subsequent casting of the mixtures into 40 × 40 × 160 mm prismatic molds were carried out in accordance with EN 196-1 [38]. The consistency and fresh density were evaluated in accordance with EN 1015-3 [37] and EN 1015-6 [39], respectively.

The sample designations for mixtures containing heavy metal-contaminated recycled aggregate are as follows:

- R—reference mixture containing only the reference aggregate, without heavy metals and without influence on cement setting time;
- RJ—mixture containing a combination of reference and recycled aggregate;
- Number (e.g., 25)—indicates the volumetric replacement percentage of recycled aggregate in the mixture;
- J—mixture containing only the recycled aggregate.

### 2.3. Setting Time and Strength Development

The microstructural development of cementitious composites is strongly time-dependent and occurs in several stages governed by the formation of various cement hydration products. Furthermore, the time-dependent velocity of ultrasonic waves directly correlates with the amount of solid phase present in the microstructure. Consequently, a higher ultrasonic pulse velocity indicates a more developed microstructure. Measuring the propagation of longitudinal ultrasonic waves is therefore a reliable method for determining the setting time of cementitious mixtures.

The setting time of the mixtures was determined using a Proceq PL-200 (produced by Proceq, Screening Eagle GmbH, Schwerzenbach, Switzerland) ultrasonic device equipped with 50 kHz longitudinal transducers. Fresh mixtures were cast into thermally insulated molds with dimensions of 10 × 10 × 10 cm. Measurements were taken at 5 min intervals. The initial setting time was defined as the time when the pulse velocity reached 800 m/s, in accordance with the literature [40]. Additionally, the onset and end of the accelerated setting phase were identified using the first derivative of the ultrasonic pulse velocity curve.

### 2.4. Temperature Evolution

During the formation of cement hydration products, reaction energy is released in the form of heat, resulting in a temperature increase within the cementitious mixture. The highest temperature typically develops at the center of the specimen. When additives that influence the setting time are incorporated, a time shift in the temperature peak can be expected. In this study, the temperature evolution of the mixtures was monitored using Ni/Cr thermocouples (Almemo, Ahlborn Mess– und Regelungstechnik GmbH, Waldkraiburg, Germany), with measurements recorded at 1 min intervals. Samples were cast into thermally insulated molds measuring 10 × 10 × 10 cm. This mold size was selected to ensure sufficient thermal mass for measurable temperature development, as smaller specimens may dissipate heat too rapidly to provide reliable data. Ambient temperature was maintained at 22 ± 1 °C. The recorded temperature profiles were further used to validate the results obtained from the ultrasonic measurements.

### 2.5. Compressive and Flexural Strength

The compressive and flexural strengths of the cementitious composites were determined on 40 × 40 × 160 mm prisms in accordance with EN 196-1 [38] after 1, 3, and 7 days of curing. All prisms were moist-cured. After casting, they remained in their molds for 24 h at >95% relative humidity, after which they were demolded and stored fully submerged in tap water from the Ljubljana public supply until testing. Flexural strength tests were carried out using an MTS Exceed Model E43 testing machine (by MTS Systems Corporation, Eden Prairie, MN, USA) with a load capacity of 50 kN and a loading rate of 0.25 mm/min. Compressive strength was measured using a hydraulic compression testing machine with a total load capacity of 5000 kN, equipped with a 500 kN load cell for precise measurement.

Flexural strength results are presented as the average of three measurements, while compressive strength results represent the average of six specimens. All results were statistically analyzed using one-way ANOVA followed by Tukey HSD post hoc testing and are reported together with their corresponding standard deviations.

### 2.6. X-Ray Diffraction Analysis (XRD)

X-ray diffraction (XRD) analysis was used to identify newly formed phases that are, or could be, a consequence of the heavy metals, present in the aggregate. Diffractograms were recorded using a Cu K⍺_1_ radiation source over a 2θ range of 5–80°. Bruker Topas software version 5 was employed for Rietveld refinement.

Prior to XRD analysis, fresh samples were dried at 40 °C and 10% relative humidity (RH). These drying conditions were selected to prevent the degradation of metastable phases while ensuring relatively rapid moisture removal. Sample annotations therefore include the drying time, as cement hydration continues even with a minimal amount of residual water.

The XRD analysis was performed on two J samples: one immediately after mixing (annotated as 3 h, including drying time) and another after 1 day of curing (also including drying time).

### 2.7. SEM-EDS

Scanning electron microscopy coupled with energy-dispersive X-ray spectroscopy (SEM-EDS) was used to examine the microstructural development of the cementitious mixtures and to identify possible new phases formed in the presence of recycled aggregate. Two samples, identical to those analyzed by XRD, were examined:

- Immediately after mixing (3 h, including drying time), and;
- After 1 day of curing.

Measurements were performed on thin polished sections. A Thermo Scientific Apreo 2 scanning electron microscope (by Thermo Fischer Scientific, Waltham, MA, USA) equipped with an EDS detector (from Oxford Instruments, Abingdon, UK) was used for the analyses. Images and elemental maps were processed using Oxford Instruments AZtec software version 6.1.

### 2.8. Thermodynamic Model

The GEMS software version 3 (by Refs. [41,42,43]) coupled with the Cemdata18 database [44] was used to verify the results obtained from the XRD analysis. The Parrot and Killoh model [45] was applied to simulate the setting process of the cementitious mixtures.

The following input assumptions were used: (1) The model incorporated the measured oxide compositions of cement and recycled aggregate. (2) Dominant carbonate phases (dolomite, calcite) were intentionally excluded to avoid artificially suppressing dissolved heavy-metal concentrations, which would dominate early-age equilibria and obscure the behavior of Zn-, Pb-, and Fe-bearing species. (3) The water content was fixed to match the experimental w/c ratio. (4) All relevant sulfate, silicate, hydroxide, and heavy-metal species predicted to form under alkaline conditions were included.

The thermodynamic model has certain limitations, particularly regarding the accuracy of the predicted product concentrations. Nevertheless, it also allows the prediction of ionic species present in the pore solution, which, at higher concentrations, may lead to the crystallization of new phases or reactions with other available species. This modeling approach provides deeper insight into the mechanisms occurring in cement composites incorporating heavy metal-contaminated recycled aggregate.

## 3. Results

### 3.1. Aggregate and Cement Characterization

**Figure 1:**
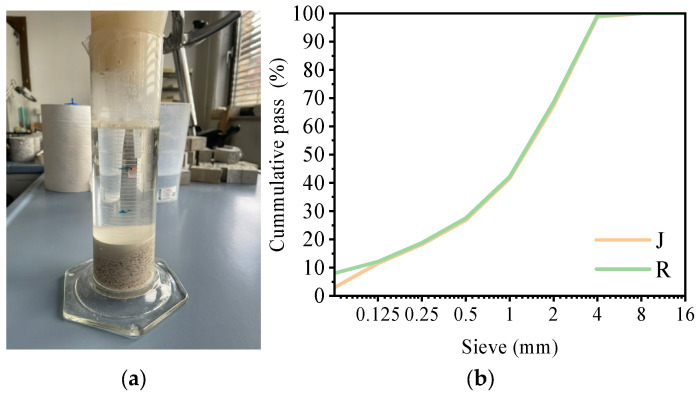
Properties of the aggregates: (**a**) absence of organic matter in the recycled aggregate (J); (**b**) grain size distribution of the reference (R) and recycled (J) aggregates.

No organic matter was detected in the recycled aggregate, as no coloration of the 3% NaOH solution was observed (Figure 1a). The particle size distribution of the selected aggregates was comparable (Figure 1b). The SSD densities of aggregates R and J were 2850 kg/m^3^ and 2730 kg/m^3^, respectively, while their water absorption values were 0.5% and 0.7%, respectively. The XRD analysis of the recycled aggregate revealed high concentrations of metal sulfides and sulfates (see Table S1). The presence of metal sulfides indicates that some unreacted or unprocessed ore residues may have been mixed with chemically processed waste.

The pH of the recycled aggregate was 8.1, while that of the reference aggregate was 9.0. The slightly lower pH of the recycled aggregate indicates a mildly more acidic behavior compared to the reference aggregate. An acidic environment is typically used to oxidize metal sulfides into sulfates, during which galvanic reactions occur and dissolved metal ions are precipitated using various chemical agents [13,14].

According to the XRD analysis, the CEM I binder consisted of 62.36% alite, 8.62% belite, 11.66% brownmillerite (C_4_AF), 3.26% C_3_A, 11.53% calcite, and 1.05% gypsum.

### 3.2. Fresh-State Properties of Cementitious Mixtures

The fresh-state properties of the cementitious mixtures are presented in Table 1.

The flowability of the mixtures increased with the proportion of recycled aggregate, to the extent that in the J mixture, a reduction in the superplasticizer (SP) dosage was required to maintain the stability of the fresh mixture. At the SP dosage of 0.6%, mix J exhibited segregation and increased bleeding. The SP content was reduced to 0.45% to achieve a stable mixture while maintaining the target w/c ratio. The density of the fresh mixtures was not significantly affected by replacing the natural aggregate (R) with recycled aggregate (J).

### 3.3. Determination of Setting Times of Mixtures Using the Ultrasound Technique

**Figure 2:**
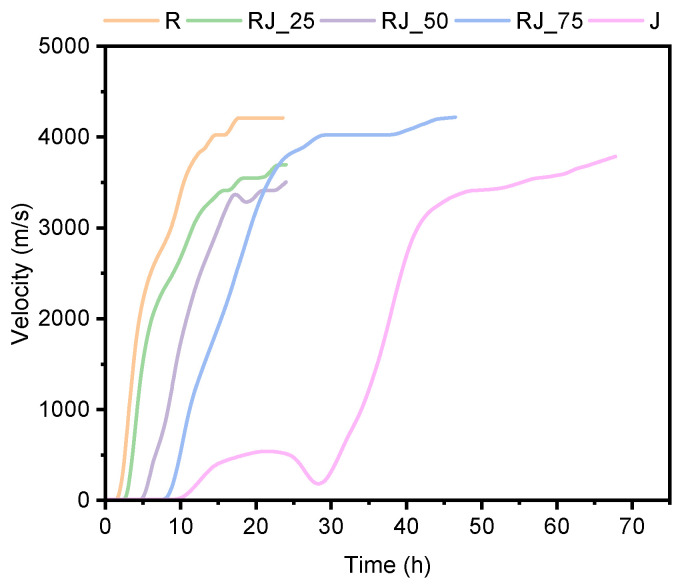
Ultrasonic pulse velocity (UPV) as a function of time for the tested mixtures.

The results of the ultrasonic pulse velocity (UPV) measurements are presented in Figure 2.

An increased proportion of recycled aggregate caused a progressive delay in the onset of setting. Mixtures RJ_25, RJ_50, RJ_75, and J exhibited delays of approximately 1 h, 6 h, 7 h, and 28 h, respectively, compared to the reference mixture R. For mixture J, no structural integrity was observed during the first 30 h, although ultrasonic data indicated the presence of a metastable microstructure. This metastable phase persisted for nearly 19 h (from 10 h to 29 h), after which a sharp increase in UPV was recorded, corresponding to the formation of C–S–H products and consolidation of the microstructure.

The slope of the initial linear region of the UPV–time curve was comparable for mixtures R and RJ_25, suggesting that the fundamental hydration mechanism of cement was not altered by contaminants present in the recycled aggregate. In contrast, mixtures RJ_50 and RJ_75 exhibited slightly lower but still comparable slopes, indicating a reduced rate of solid-phase formation. The distinctly different behavior of mixture J confirms the strong influence of the recycled aggregate on early-age hydration kinetics.

The first derivative of the UPV–time curve (Figure S1) revealed additional inflection points in mixtures with higher recycled aggregate contents (50% and 100%), indicating temporary stabilization of secondary or heavy-metal–influenced intermediate phases.

Overall, the setting time period (defined as the time between the initial and final setting, marked by the left and right dotted lines in Figure S1) was approximately 7–8 h for all tested mixtures. This indicates that the incorporation of recycled aggregate does not significantly affect the setting period itself, but primarily delays the initial setting time and consequently the early microstructure development.

### 3.4. Hydration Temperature Evolution

**Figure 3:**
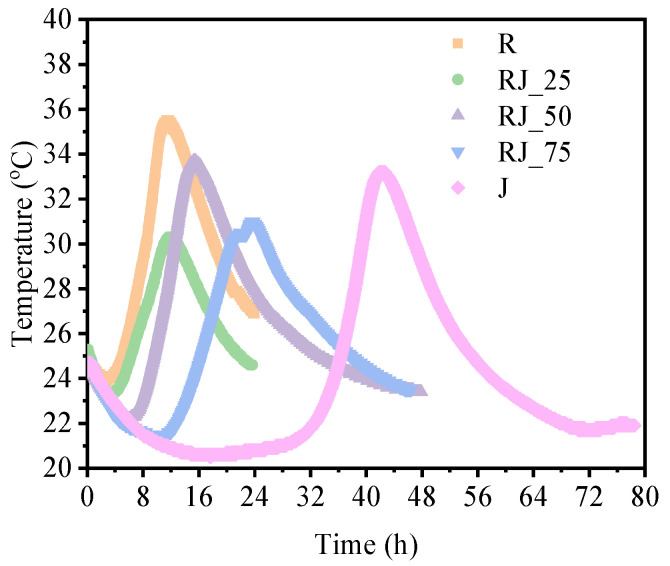
Temperature evolution as a function of time for the tested mixtures.

The temperature evolution of the mixtures showed a strong correlation with the results obtained from the ultrasonic measurements. The highest temperature in each mixture was recorded near the final setting time (Figure 3). Moreover, the temperature development was influenced by the presence of heavy metals in the recycled aggregate.

The onset of temperature rise following the dormant phase occurred at approximately 3.67 h (R), 4.17 h (RJ_25), 6.90 h (RJ_50), 12.13 h (RJ_75), and 31.45 h (J). These values differ slightly from the UPV-determined initial setting times; however, the thermal curves reliably tracked the trends in hydration delay.

### 3.5. Flexural and Compressive Strength

**Figure 4:**
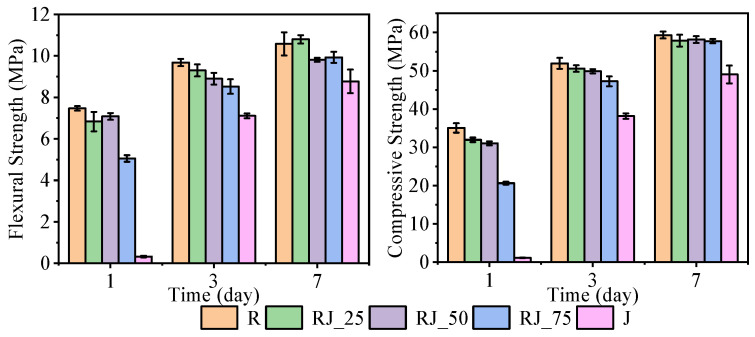
Flexural (**left**) and compressive (**right**) strengths of the tested mixtures after 1, 3 and 7 days.

The flexural and compressive strengths of the mixtures after 1, 3, and 7 days are presented in Figure 4.

After 1 day, the flexural strengths of the reference mixture (R) and mixtures RJ_25 and RJ_50 were comparable, with values of around 7 MPa. Mixtures containing a higher proportion of recycled aggregate exhibited a marked reduction in flexural strength, decreasing to approximately 5 MPa for RJ_75 and to below 0.5 MPa for J, which is consistent with the observed delay in setting. After 3 days, the flexural strengths of mixtures R through RJ_50 remained comparable, mostly ranging between 9 and 9.5 MPa, whereas RJ_75 and J showed lower values than the reference. After 7 days, mixtures R through RJ_75 achieved statistically comparable flexural strengths, predominantly between 10 and 11 MPa. The lowest flexural strength at this age was recorded for mixture J, with values between 8 and 9 MPa.

Greater differences were observed in compressive strength. After 1 day, mixture R reached 35 MPa, while RJ_25 and RJ_50 both achieved approximately 32 MPa. Mixture RJ_75 reached 20 MPa, and J was unable to withstand any compressive load at this age due to incomplete setting. After 3 days, mixtures R, RJ_25, and RJ_50 developed compressive strengths of around 50 MPa, although the strength of RJ_50 was statistically significantly lower than that of R. Mixture RJ_75 reached approximately 90% of the reference value. Mixture J exhibited the greatest delay in strength development, with strengths remaining below 40 MPa by day 3. After 7 days, all mixtures except J achieved comparable compressive strengths of about 58 MPa, while mixture J reached values close to 50 MPa.

These results demonstrate that the incorporation of recycled aggregate affects the early-age strength development, particularly within the first 24 h, due to the delayed hydration observed in the UPV and temperature analyses. The presence of heavy metals likely interferes with the early formation of stable hydration products, leading to the temporary development of metastable phases with lower stiffness and cohesion. As hydration progresses, however, the differences diminish, and the long-term strength of mixtures containing recycled aggregate approaches that of the reference mixture.

### 3.6. XRD Analysis

**Figure 5:**
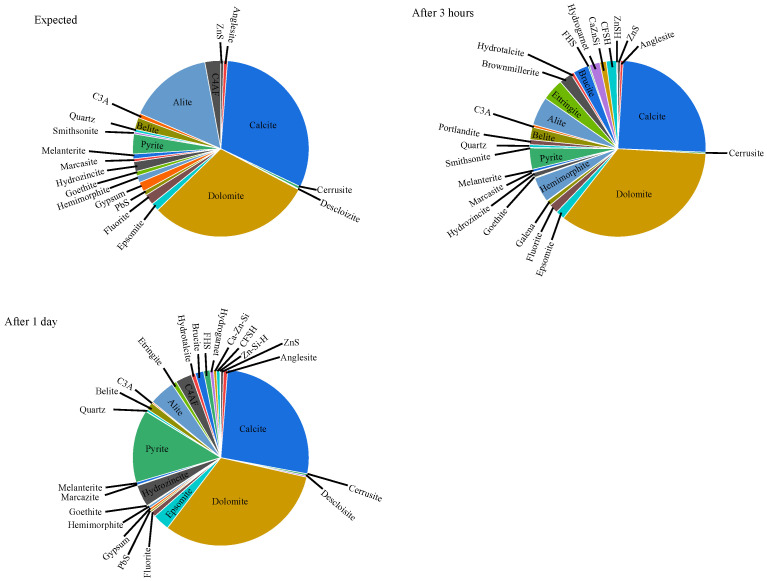
XRD analysis of mixture J.

The XRD results of mixture J after 3 h and 24 h, together with the expected initial phase assemblage, are presented in Figure 5. Comparison between the measured and expected compositions provides valuable insight into the processes occurring within the cementitious system. The expected initial composition was calculated based on the XRD-quantified mineralogy of the cement and recycled aggregate, weighted according to their proportions in the mixture.

The analyses revealed that ZnS remains stable in the alkaline cementitious environment. However, after 3 h, the concentrations of hydrozincite and smithsonite decreased, while the concentration of hemimorphite increased. The reduction in smithsonite (ZnCO_3_) and hydrozincite alongside an increase in hemimorphite suggests that Zn^2+^ ions reacted with SiO_3_^2−^ ions released from alite dissolution. This reaction likely occurred under oxidizing conditions in the presence of galena, anglesite, and smithsonite. Complex metal silicate species—primarily containing Ca, Fe, Zn, and Si—were most abundant after the first 3 h. After 24 h, their concentrations decreased, accompanied by the formation of FeS_2_, gypsum, and hydrozincite. The appearance of FeS_2_ indicates a transition toward reducing conditions within the cement matrix.

Galena (PbS) is known to dissolve in alkaline environments, releasing SO_4_^2−^ and HPbO_2_^−^ ions. After 24 h, these ions recombined to form PbSO_4_, once the concentration of SO_4_^2−^ ions became sufficiently high. Similar behavior was observed for cerussite, while Pb^2+^ ions did not appear to interact with major cement phases such as alite, belite, brownmillerite, or C_3_A.

Among the metal species detected, iron compounds exhibited the highest concentrations compared to lead and zinc species. The dissolution of iron-bearing phases released Fe^2+^ ions, which subsequently reacted with SiO_3_^2−^ species to form iron silicate complexes such as Fe_11_Si_4_(H_4_O_9_)_3_ and hydrogarnet. The brownmillerite (C_4_AF) phase itself remained largely unhydrated after 24 h, as its hydration typically progresses after several days.

After 24 h, the formation of hydrotalcite, brucite, and epsomite was observed. The Mg^2+^ ions required for their formation likely originated from the cement itself, from the regeneration of epsomite, and to a lesser extent from dedolomitization processes (alkali–carbonate reaction, ACR). The formation of epsomite (MgSO_4_·7H_2_O) appears thermodynamically more favorable than that of anglesite, with the necessary SO_4_^2−^ ions supplied by gypsum, melanterite, or by ettringite recrystallization.

The concentrations of alite and belite decreased due to hydration, with belite hydrating more slowly, as expected. The concentration of ettringite after 3 h remained relatively low despite the high availability of SO_4_^2−^ ions, indicating that sulfate-bearing phases were preferentially involved in reactions with metallic species during early hydration.

### 3.7. SEM-EDS Analysis

**Figure 6:**
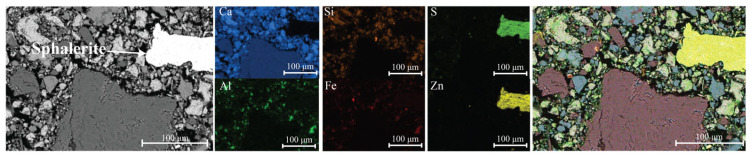
SEM-EDS micrograph of mixture J after 3 h. A sphalerite particle appears as a bright (white) inclusion within the binder matrix.

The SEM-EDS analysis of mixture J confirmed the presence of zinc-bearing species within the recycled aggregate (Figure 6). After 3 h, the microstructure was not yet developed, as visible voids between the aggregate particles and the cement matrix were still present. The lead-bearing species were mostly below the detection limit; however, traces of lead were detected within the aggregate (Figure 6).

**Figure 7:**
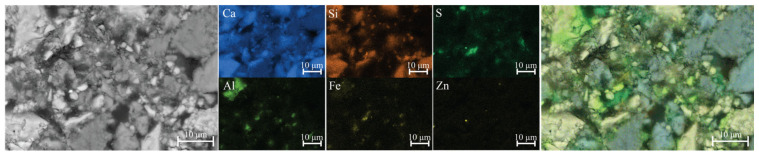
SEM-EDS analysis of mixture J after 1 day. The microstructure is more developed, with sulfur- and zinc-bearing species still present within the cement matrix.

**Figure 8:**
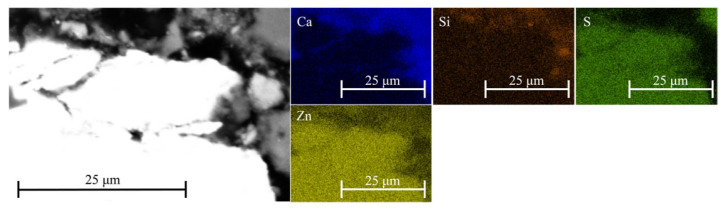
SEM-EDS analysis of mixture J after 1 day demonstrating the formation of the C-Zn-S-H phase within the microstructure.

**Figure 9:**
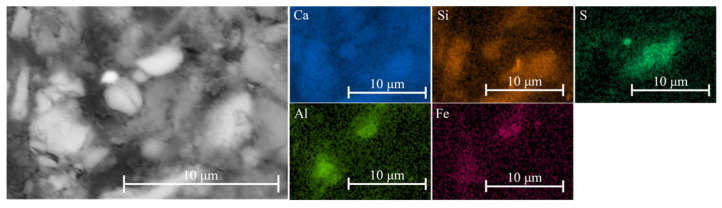
SEM-EDS analysis of mixture J after 1 day demonstrating the formation of the C–F–S–H phase within the microstructure.

The 1-day-old sample exhibited a more consolidated microstructure (Figure 7). Localized areas enriched in sulfur and silicon were observed, indicating the formation of early hydration products and secondary phases. Phases containing lead and zinc, which are expected to form under alkaline conditions, were detected only in a few instances—most likely due to their low concentration, limited stability, or transformation into amorphous or metastable products below the EDS detection threshold. Moreover, the formation of C-Zn-S-H is most likely restricted to regions in close proximity to the ZnS particle (Figure 8). In contrast, iron–silica-rich phases were more abundant in the cementitious matrix (Figure 9), consistent with the widespread presence of iron-bearing minerals in the system.

### 3.8. Phase Prediction Using Thermodynamic Model

**Figure 10:**
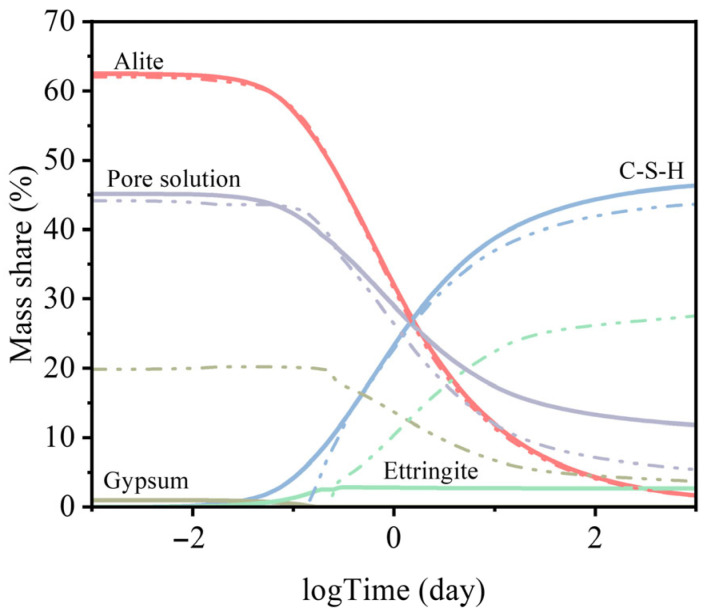
Thermodynamic model of the setting mechanism for mixtures containing reference (solid line) and recycled (dash–dot line) aggregates. Based on the differences in the evolution of C–S–H, gypsum, ettringite, and the pore solution, the model successfully predicts the influence of heavy metals on the initial setting time.

Figure 10 presents the results of thermodynamic modeling of cement hydration for the reference mixture (R) (solid line) and the mixture containing recycled aggregate (J) (dash–dot line). For clarity, only the dominant hydration products are shown, although all relevant phases were included in the calculations.

The model highlights clear differences between the hydration behavior of the reference cementitious mixture and the mixture containing recycled aggregate. In the case of mixture J, the formation of C–S–H gel was delayed by approximately 2.5 h compared to the reference. Additionally, the ettringite concentration reached about 25 mass%, due to enhanced gypsum formation. This suggests that an increased availability of sulfate ions—likely originating from the recycled aggregate—could have contributed to a sulfate-related retardation of setting in the mixture J.

**Figure 11:**
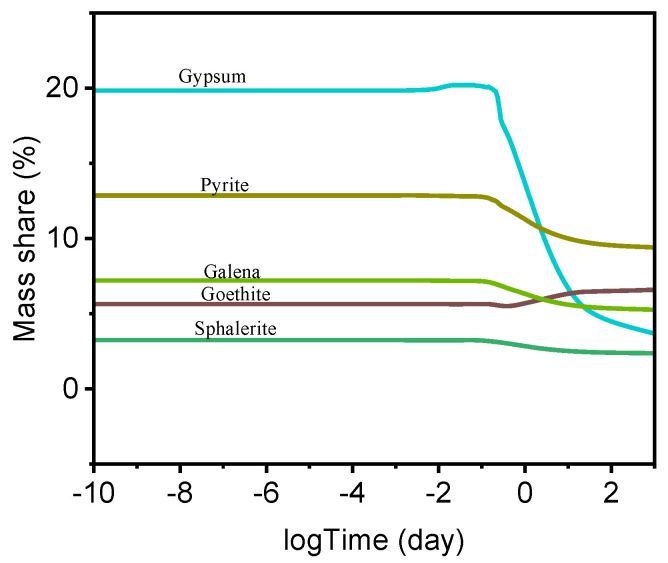
Thermodynamic model predicting the dissolution and crystallization of metal species originating from the recycled aggregate.

Moreover, the thermodynamic model can also predict the interactions between the hydrated cement paste and heavy metals originating from the recycled aggregate. Figure 11 illustrates the dissolution behavior of heavy metals in the alkaline cementitious environment. The concentration of goethite increased during hydration as a result of the elevated availability of Fe^2+^ species and OH^−^ ions. The model also predicts the dissolution of wurtzite (ZnS, hexagonal crystal structure), which would release Zn^2+^ ions into the pore solution while simultaneously generating S^2−^ that may subsequently oxidize to SO_4_^2−^ under the prevailing oxidative conditions.

**Figure 12:**
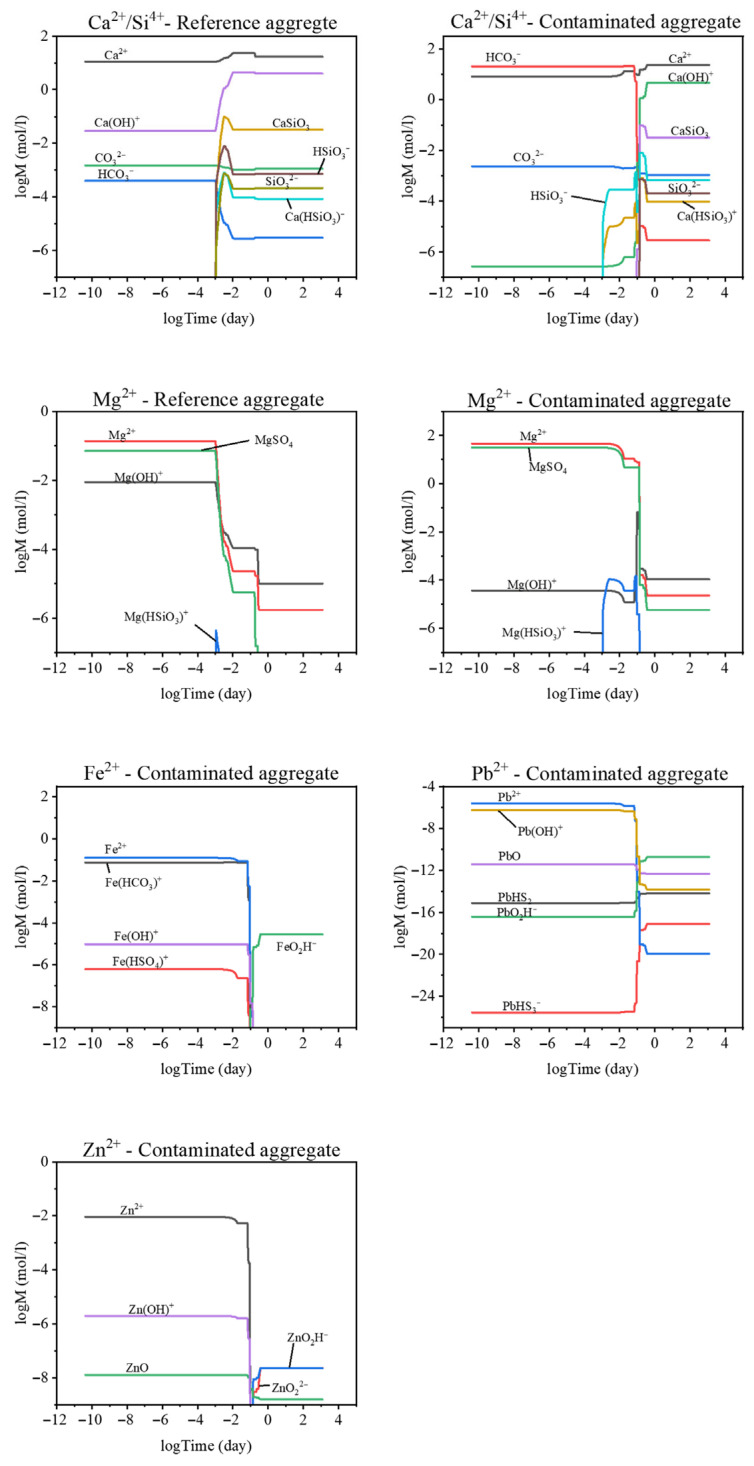
Comparison of ionic species in the pore solution of the reference mixture and the mixture containing recycled aggregate.

Furthermore, the pore solution of the heavy metal-contaminated cement binder was modeled (Figure 12). The concentrations of Ca and Si species suggest a higher proportion of HCO_3_^−^ relative to Ca^2+^ compared with normal cement hydration. In the system containing the recycled aggregate, the initial concentration of Ca(OH)^+^ ions was very low (≈10^−6^ mol/L) but reached values comparable to the reference mixture after 7.6 h. For comparison, the peak concentration of Ca(OH)^+^ ions in the reference mixture occurred after only 0.24 h.

For Mg species, the recycled aggregate mixture exhibited a lower initial concentration of Mg(OH)^+^, followed by a steep increase. After one day, the concentration of Mg(OH)^+^ ions stabilized and remained higher than in the reference mixture. The delayed formation of Mg(OH)^+^, and consequently hydrotalcite, can be attributed to interactions with dissolved heavy metals present in the pore solution. At the same time, the concentration of Mg(HSiO_3_)^+^ was higher than in the reference system, and its formation coincided with the consumption of Mg^2+^ and MgSO_4_.

In alkaline environments, iron forms several aqueous species, including Fe^2+^, Fe(HCO_3_)^+^, Fe(OH)^+^, Fe(HSO_4_)^+^, and FeO_2_H^−^. All iron species except FeO_2_H^−^ were consumed after approximately 2.4 h, while the concentration of FeO_2_H^−^ increased thereafter. Lead in the pore solution occurred primarily as Pb^2+^, Pb(OH)^+^, PbO, PbHS_2_, PbO_2_H^−^, and PbHS_3_^−^ ions. The highest concentrations, on the order of 10^−6^ M, were found for Pb^2+^ and Pb(OH)^+^, which were several orders of magnitude lower than the dominant iron species. After 2.4 h, the decrease in Pb^2+^ and Pb(OH)^+^ concentrations suggests precipitation or reaction of lead-containing phases.

Zinc species exhibited higher concentrations than those of lead but remained roughly an order of magnitude lower than the iron species. Within the concentration range of 10^−2^–10^−8^ M, the main zinc-bearing species identified were Zn_2_O, Zn(OH)^+^, ZnO, ZnO_2_^2−^, and ZnO_2_H^−^. Similarly to the behavior of other metal species, the concentrations of Zn^2+^ and Zn(OH)^+^ decreased after 2.4 h, accompanied by the formation of ZnO_2_H^−^ and ZnO_2_^2−^ at concentrations around 10^−7.5^ M.

According to the thermodynamic model, the dissolution of metallic species and the concurrent formation of gypsum created reducing conditions within the system, as further evidenced by the predicted formation of pyrite (FeS_2_).

## 4. Discussion

The XRD analysis of the recycled aggregate revealed the presence of various metal sulfides and sulfates, including sphalerite, galena, pyrite, smithsonite, epsomite, hemimorphite, and melanterite, with concentrations ranging from 0.3% to 2.8%. Among these, iron sulfides were the most abundant, representing residual phases not fully processed during ore extraction. According to the literature, the coexistence of iron, lead, and zinc sulfides can significantly hinder efficient ore separation and recovery during flotation and roasting processes [46,47].

The extraction of lead and zinc ores typically occurs under oxidizing and high-temperature conditions, promoting the oxidation of metal sulfides into water-soluble sulfate compounds. Such conditions may also contribute to thermal decomposition of dolomite and the subsequent formation of epsomite (MgSO_4_·7H_2_O). The relatively high concentrations of sulfates and sulfides identified in the recycled aggregate explain its slightly lower pH compared to the reference aggregate. As noted by Ref. [48], the pH of an aggregate provides a reliable indication of its mineralogical composition.

An increasing proportion of recycled aggregate enhanced flowability of the mixtures, with flow values rising from 131.5 mm (R), thorough 152.5 mm (RJ_25) and 173.5 mm (RJ_50), up to 214.0 mm (RJ_75). The lower flow value of mixture J (196.5 mm) resulted from its reduced superplasticizer dosage, which was decreased from 0.60% to 0.45% to ensure the stability of mixture J. This reduction in SP content may affect the onset and progression of cement paste setting, shifting the initial setting time earlier. Consequently, the UPV values obtained for mixture J lie on the safe side. With the same SP dosage (0.60%), a longer initial setting time would be expected. Enhanced flowability with increasing recycled aggregate content may be attributed to the constituents within the recycled aggregate that improve particle dispersion in the cement paste—a macroscopic effect analogous to that produced by superplasticizers. Moreover, the results suggest that the proportion of free water available to govern mixture consistency may also increase with higher recycled aggregate content, which can further contribute to improved flow. Comparable observations were made by Zhong et al. (2025) [49], who reported improved workability when using coarser sintered aggregates derived from heavy metal sludge, attributing the effect primarily to particle morphology. Conversely, Li et al. (2023) [15] reported the opposite effect when incorporating recycled concrete powders, where the higher porosity of the recycled material reduced the workability of the mixture. These findings demonstrate that the influence of recycled materials on flowability depends strongly on surface texture, porosity, and chemical composition.

In contrast, increasing the proportion of recycled aggregate significantly prolonged the setting time of the mixtures. UPV measurements of mixture J revealed the development of a metastable microstructure after approximately 12 h (Figure 2), indicating delayed formation of a continuous solid network. Early-age XRD analysis confirmed the presence of ettringite (4%) and hemimorphite (4.6%), suggesting that sulfate- and zinc-bearing phases formed preferentially during the initial hydration period.

In addition to these crystalline phases, both modeling and experimental data indicate the possible formation of unstable mixed phases, such as C–F–S–H, Ca–Zn–Si, F–H–S, and Zn–S–H complexes. These transient species exhibit a high affinity for silicate ions, diverting them from conventional C–S–H gel formation and thereby slowing microstructural development. Concurrently, dissolution and reprecipitation of zinc and lead compounds alter the ionic balance of the pore solution, further modifying hydration kinetics.

After one day, the XRD results showed a reduction in metal silicate phases accompanied by an increase in FeS_2_, anglesite (PbSO_4_), epsomite (MgSO_4_·7H_2_O), gypsum, and hydrozincite (Zn_5_(CO_3_)_2_(OH)_6_). This transformation indicates a progressive shift from early metastable phases toward more stable sulfate and carbonate species, consistent with the observed delay in setting and early strength development.

The results of the flexural and compressive tests demonstrate that statistically significant mechanical effects arise primarily at high replacement levels of recycled aggregate (≥50 vol.%) and are most pronounced within the first 24–48 h. At lower replacement levels (≤25 vol.%), the recycled aggregate does not compromise structural performance, whereas at higher levels the early inhibition of hydration—likely induced by interactions between heavy-metal cations and the forming hydrates—can delay strength development but does not prevent substantial recovery at later ages. In particular, mixture J showed no structural integrity after one day, indicating that the early microstructure was too weak to resist external stresses. SEM analysis of the 1-day-old sample confirmed the presence of a continuous but weakly bonded matrix, characterized by poorly crystalline metal–silicate phases instead of conventional C–S–H. These metal–silicate complexes typically form as solid solutions with limited stiffness and therefore do not contribute effectively to mechanical interlocking or load transfer. Even at relatively low concentrations—only a few percent—the metallic species introduced via the recycled aggregate clearly interacted with early hydration, as evidenced by the UPV results, the delayed setting, and the suppressed 1-day mechanical strengths. At later ages, these effects became markedly less pronounced. By 7 days, the strengths of mixtures R through RJ_75 were statistically comparable, while mixture J—although still weaker—showed substantial recovery. This confirms that the adverse influence of the recycled aggregate on hydration is primarily limited to the first 24–48 h and does not prevent the subsequent formation of stable C–S–H phases.

The thermodynamic model provided additional insight into the hydration processes of the cementitious system and successfully predicted the delayed setting time observed experimentally. However, the model could not capture the formation of certain hydration products associated with heavy metal cations, likely because these phases are metastable or otherwise outside the equilibrium assumptions inherent to thermodynamic modeling. Nonetheless, such modeling remains a valuable predictive tool for identifying potential long-term risks, such as delayed sulfate attack, which may arise from the secondary gypsum formation originating from iron and magnesium species. These processes could have a significant impact on the long-term durability and mechanical performance of the composite.

### 4.1. Interpretation of Reaction Pathways

According to the findings of this study, the interactions between the metal species in the recycled aggregate and the cement matrix can be summarized as follows:

(a) Formation of hemimorphite from hydrozincite (Equation (1)), and from smithsonite (Equation (2)), in the presence of anglesite, cerrusite, pyrite and galena in oxidizing conditions:

4Zn_5_(CO_3_)_2_(OH)_6(s)_ + 10SiO_3_^2−^_(aq)_ ⟶ 5Zn_4_Si_2_O_7_(OH)_2_·H_2_O_(s)_ + 8CO_3_^2−^_(aq)_ + 4OH^−^_(aq)_ (1)

4ZnCO_3(s)_ + 2SiO_3_^2−^_(aq)_ + 4OH^−^_(aq)_ ⟶ Zn_4_Si_2_O_7_(OH)_2_·H_2_O_(s)_ + 4CO_3_^2−^_(aq)_ (2)

(b) Formation of mixed metal complexes (Ca–Al–Fe–Si, Ca–Zn–Si, Zn–Si) (Equations (3)–(5)):

2Ca^2+^_(aq)_ + 2Al(OH)^4−^_(aq)_ + Fe(OH)^4−^_(aq)_ + 3SiO_3_^2−^_(aq)_ + 2H_2_O_(l)_ ⟶ Ca_2_Al_2_FeSi_3_O_12_·2H_2_O_(s)_ (3)

Ca(OH)^+^_(aq)_ + SiO_3_^2−^_(aq)_ + Zn^2+^_(aq)_ + OH^−^_(aq)_ ⟶ CaZnSiH_2_O_5(s)_ (4)

2Zn(OH)^+^_(aq)_ + SiO_3_^2−^_(aq)_ ⟶ Zn_2_SiH_2_O_5(aq)_ (5)

(c) Dissolution of melanterite with the release of SO_4_^2−^ (Equation (6)) and formation of epsomite from brucite under reducing conditions (Equation (7)):

FeSO_4_·H_2_O_(s)_ + 2OH^−^_(aq)_ ⟶ Fe^2+^_(aq)_ + SO_4_^2−^_(aq)_ + 2H_2_O_(l)_ (6)

Mg(OH)_2(s)_ + SO_4_^2−^_(aq)_ + 3H_2_O_(l)_ + 2H_3_O^+^_(aq)_ ⟶ MgSO_4_·7H_2_O_(s)_ (7)

(d) Formation of pyrite (FeS_2_) under reducing conditions in the presence of excess SO_4_^2−^ and Fe^2+^ ions (Equation (8)), where sulfate must first be reduced to sulfide species:

SO_4_^2−^_(aq)_ + Fe^2+^_(aq)_ ⟶ FeS_2(s)_ + 4O_2(aq)_ (8)

(e) Formation of gypsum from SO_4_^2−^ ions originating from anglesite, epsomite and melanterite dissolution
(f) Disintegration of hemimorphite, release of SiO_3_^2−^ ions, formation of hydrozincite, renewal of oxidizing state and formation of C–S–H gel.

The delayed setting time can thus be attributed to the formation of metastable metal-bearing species that temporarily lower the oxidation potential of the mixture. Under these reducing conditions, the rate of hydration decreases, metastable phases gradually decompose, and metal sulfides (e.g., FeS_2_) form. As the system re-establishes oxidizing conditions through sulfide oxidation and secondary sulfate formation, the environment once again becomes favorable for the C–S–H gel formation and the progression of normal hydration reactions.

### 4.2. Practical Implications and Future Research Needs

The results of this study show that recycled aggregate derived from Pb–Zn mining waste can be used in cement-based composites, but only within a restricted substitution range. A replacement level of 25 vol.% proved optimal, as it did not impair flowability, setting, early hydration, or early-age strength, indicating that limited incorporation of such material is feasible for practical applications without compromising performance in the fresh or early hardened state.

From a long-term perspective, the elevated sulfur content of the recycled aggregate may pose risks of delayed sulfate attack or secondary gypsum formation, which can induce microcracking and increase susceptibility to environmental degradation [50,51,52]. Furthermore, although heavy metals remained immobilized during the tested early stages, their potential release under severe deterioration, microcracking, or environmental exposure remains insufficiently understood. This represents a critical knowledge gap for assessing environmental safety and full life-cycle performance.

To address these uncertainties, further research should include systematic durability testing under carbonation, chloride ingress, freeze–thaw cycling, and sulfate exposure to fully evaluate resistance to long-term degradation. Equally important is the need to investigate whether microstructural deterioration could mobilize Pb or Zn over time.

## 5. Conclusions

The presence of heavy metals in recycled aggregate from Pb–Zn mine tailings significantly prolongs the setting time of cementitious composites and delays early-age strength development. This behavior is attributed to the formation of metastable metal-bearing phases that temporarily modify the redox environment and interfere with early hydration, particularly by suppressing silicate availability and delaying C–S–H formation.

Integrated ultrasonic and temperature monitoring, supported by thermodynamic modeling and XRD analysis, provided complementary insight into these mechanisms. Together, the results demonstrate that Zn-, Pb-, and Fe-bearing mineral phases alter the dissolution–precipitation balance during the earliest stages of hydration, affecting the development of a load-bearing microstructure.

Despite the observed retardation effects, heavy-metal-bearing recycled aggregate can be used effectively in concrete production when applied within appropriate concentration limits. A replacement level of 25 vol.% was identified as optimal, ensuring acceptable setting behavior and mechanical performance without compromising early hydration. Higher replacement levels, however, caused substantial delays in hydration and early-age strength development.

Before large-scale implementation, recycled aggregate from metalliferous waste streams requires thorough mineralogical and chemical characterization to ensure compatibility with cement, supplementary cementitious materials, and admixtures. Particular attention should be given to long-term durability and environmental safety, including potential risks associated with secondary sulfate formation, delayed sulfate attack, and the mobility of heavy metals under severe degradation or environmental exposure.

## Figures and Tables

**Table 1:** Properties of the fresh mixtures.

Mixture	w/c	Superplasticizer [%]	Flowability [mm]	Density [kg/m^3^]
R	0.45	0.6	131.5	2291
RJ_25	0.45	0.6	152.5	2252
RJ_50	0.45	0.6	173.5	2290
RJ_75	0.45	0.6	214	2261
J	0.45	0.45	196.5	2260

## Data Availability

The original contributions presented in this study are included in the article/Supplementary Materials, further inquiries can be directed to the corresponding author.

## Supplementary Materials

The following supporting information can be downloaded at: https://www.mdpi.com/article/10.3390/ma18245533/s1, Figure S1: Determining the initial and final setting times using the first derivative of ultrasonic pulse velocity (UPV). The local maxima observed in the first derivative correspond to distinct phases formed during cement hydration. Table S1: Mineralogical composition of the recycled aggregate J determined by XRD.

## References

[1] Mehta P.K., Monteiro P.J.M. (2013). Concrete: Microstructure, Properties, and Materials.

[2] Nearly-Zero Energy and Zero-Emission Buildings—European Commission. https://energy.ec.europa.eu/topics/energy-efficiency/energy-efficient-buildings/nearly-zero-energy-and-zero-emission-buildings_en?utm_source=chatgpt.com.

[3] Wang B., Yan L., Fu Q., Kasal B. (2021). A Comprehensive Review on Recycled Aggregate and Recycled Aggregate Concrete. Resour. Conserv. Recycl..

[4] Xing W., Tam V.W., Le K.N., Hao J.L., Wang J. (2022). Life Cycle Assessment of Recycled Aggregate Concrete on Its Environmental Impacts: A Critical Review. Constr. Build. Mater..

[5] Tam V.W.Y., Soomro M., Evangelista A.C.J. (2018). A Review of Recycled Aggregate in Concrete Applications (2000–2017). Constr. Build. Mater..

[6] Le H.B., Bui Q.B. (2020). Recycled Aggregate Concretes—A State-of-the-Art from the Microstructure to the Structural Performance. Constr. Build. Mater..

[7] Kovacs R., Shamass R., Limbachiya V. (2025). Multi-Recycling of Different Concrete Products: Effects on Recycled Aggregate’s Physical Characteristics and Compressive Strength. J. Build. Eng..

[8] Alkhteeb L., Dawood M.B. (2025). The Effect of Recycled Aggregate on Properties of Concrete: A Review. Hybrid. Adv..

[9] Wongkvanklom A., Posi P., Wongsa A., Zaetang Y., Yodsudjai W. (2025). Enhanced Strength Reduced Modulus High Calcium FA Geopolymer Concrete Containing Recycled Aggregate Concrete and Portland Cement. Clean. Waste Syst..

[10] Martins N.P., Srivastava S., Simão F.V., Niu H., Perumal P., Snellings R., Illikainen M., Chambart H., Habert G. (2021). Exploring the Potential for Utilization of Medium and Highly Sulfidic Mine Tailings in Construction Materials: A Review. Sustainability.

[11] Huang B., Lai H., Deng J., Xu H., Fan G. (2019). Study on the Interaction between Galena and Sphalerite during Grinding Based on the Migration of Surface Components. ACS Omega.

[12] Lead. https://www.essentialchemicalindustry.org/metals/lead.html#.

[13] Pashkevich M., Alekseenko A., Nureev R. (2023). Environmental Damage from the Storage of Sulfide Ore Tailings. J. Min. Inst..

[14] Maltrana V., Morales J. (2023). The Use of Acid Leaching to Recover Metals from Tailings: A Review. Metals.

[15] Li J., Zhan B., Gao P., Hu L., Qiao M., Sha H., Yu Q. (2023). Effects of Recycled Concrete Powders on the Rheology, Setting and Early Age Strength of Cement Paste. Constr. Build. Mater..

[16] Xia Q., Bai Y., Han L. (2025). Discussion on the Applicability and Mechanism of Foamed Geopolymer Concrete Used for Cadmium Heavy Metals Solidification/Stabilization. Constr. Build. Mater..

[17] Keppert M. (2018). Retarding Action of Various Lead (II) Salts on Setting of Portland Cement. Proceedings of the Key Engineering Materials.

[18] Massarweh O., Maslehuddin M., Al-Dulaijan S.U., Shameem M. (2021). Performance Evaluation of Heavy Oil Fly Ash as a Retarder of Portland Cement Hydration. J. Build. Eng..

[19] Garg N., White C.E. (2017). Mechanism of Zinc Oxide Retardation in Alkali-Activated Materials: An: In Situ X-Ray Pair Distribution Function Investigation. J. Mater. Chem. A Mater..

[20] Krishnaraj L., Ramesh N., Kumar R.S., George P.K. (2020). Characterization Study of Zinc Sulphate’s Influence and Retarding Mechanism with Coarser and Finer Fly Ash Particles in Concrete. KSCE J. Civil. Eng..

[21] Silatikunsatid T., Jaitanong N., Narksitipan S. (2018). A Study on Influence of Zinc Oxide in Cement Composite Materials. Proceedings of the Key Engineering Materials.

[22] Nedyalkova L., Lothenbach B., Renaudin G., Mäder U., Tits J. (2019). Effect of Redox Conditions on the Structure and Solubility of Sulfur- and Selenium-AFm Phases. Cem. Concr. Res..

[23] Wieland E., Miron G.D., Ma B., Geng G., Lothenbach B. (2023). Speciation of Iron(II/III) at the Iron-Cement Interface: A Review. Mater. Struct..

[24] Nonat A. (2004). The Structure and Stoichiometry of C-S-H. Cem. Concr. Res..

[25] Taylor H.F. (1997). Cement Chemistry.

[26] Chen X., Guo Y., Ding S., Zhang H., Xia F., Wang J., Zhou M. (2018). Utilization of Red Mud in Geopolymer-Based Pervious Concrete with Function of Adsorption of Heavy Metal Ions. J. Clean. Prod..

[27] Giergiczny Z., Król A., Król K. (2008). Immobilization of Heavy Metals (Pb, Cu, Cr, Zn, Cd, Mn) in the Mineral Additions Containing Concrete Composites. J. Hazard. Mater..

[28] Weiler L., Vega Garcia P., Vollpracht A. (2025). Outdoor Exposure of a Heavy Metal Doped Concrete –Measuring and Modelling of Substance Release. J. Environ. Manag..

[29] Park K.S., Zajac M., Matschei T., Vollpracht A. (2025). The Fate of Heavy Metals in Recycled Concrete Paste upon Enforced Carbonation: A Review. Resour. Conserv. Recycl. Adv..

[30] Qi Y., Wang Q., Li Z., Xiong Y., Wang C. (2025). Durability and Microstructure of Bulk Solid Waste Recycled Concrete Load-Bearing Block under Freeze-Thaw Cycles. J. Build. Eng..

[31] (2013). Tests for Chemical Properties of Aggregates. Part 1, Chemical Analysis.

[32] (2012). Tests for Geometrical Properties of Aggregates. Part 1, Determination of Particle Size Distribution: Sieving Method.

[33] (2022). Tests for Mechanical and Physical Properties of Aggregates. Part 6, Determination of Particle Density and Water Absorption.

[34] (2020). Test Method for Silica—pH Value.

[35] (2011). Cement—Part 1: Composition, Specifications and Conformity Criteria for Common Cements.

[36] (2016). RRT+ Main Phase of the Extended Round Robin Testing Programme for TU1404. Testing Protocols; TU 1404 COST ACTION; Towards the Next Generation of Standards for Service Life of Cement-Based Materials and Structures. https://www.tu1404.eu/wp-content/uploads/2017/12/RRT-Main-phase_Protocols_06112017.pdf.

[37] (1999). Methods of Test for Mortar for Masonry, Part 3: Determination of Consistence of Fresh Mortar (by Flow Table).

[38] (2016). Methods of Testing Cement, Part 1: Determination of Strength.

[39] (2007). Methods of Test for Mortar for Masonry, Part 6: Determination of Bulk Density of Fresh Mortar.

[40] Serdar M., Gabrijel I., Schlicke D., Staquet S., Azenha M. (2020). Advanced Techniques for Testing of Cement-Based Materials.

[41] Kulik D.A., Wagner T., Dmytrieva S.V., Kosakowski G., Hingerl F.F., Chudnenko K.V., Berner U. (2013). GEM-Selektor geochemical modeling package: Revised algorithm and GEMS3K numerical kernel for coupled simulation codes. Comput. Geosci..

[42] Wagner T., Kulik D.A., Hingerl F.F., Dmytrieva S.V. (2012). GEM-Selektor geochemical modeling package: TSolMod library and data interface for multicomponent phase models. Can. Mineral..

[43] Miron G.D., Kulik D.A., Dmytrieva S.V., Wagner T. (2015). GEMSFITS: Code package for optimization of geochemical model parameters and inverse modeling. Appl. Geochem..

[44] Lothenbach B., Kulik D.A., Matschei T., Balonis M., Baquerizo L., Dilnesa B., Miron G.D., Myers R.J. (2019). Cemdata18: A Chemical Thermodynamic Database for Hydrated Portland Cements and Alkali-Activated Materials. Cem. Concr. Res..

[45] Parrot L.J., Killoh D.C. (1984). Prediction of cement hydration. Br. Ceram. Proc..

[46] Aikawa K., Ito M., Segawa T., Jeon S., Park I., Tabelin C.B., Hiroyoshi N. (2020). Depression of Lead-Activated Sphalerite by Pyrite via Galvanic Interactions: Implications to the Selective Flotation of Complex Sulfide Ores. Miner. Eng..

[47] Lindsay M.B.J., Moncur M.C., Bain J.G., Jambor J.L., Ptacek C.J., Blowes D.W. (2015). Geochemical and Mineralogical Aspects of Sulfide Mine Tailings. Appl. Geochem..

[48] Wang Y., Chen J., Yan Q., Peng Y., Kong L. (2023). Quantitative Characterization of Aggregates and Their Chemical Constituents in Terms of Acid and Alkaline Indicators. Case Stud. Constr. Mater..

[49] Zhong W., Wang S., Chen Y., Ye N., Shu K., Dai R., Ba M. (2025). Effects of Heavy-Metal-Sludge Sintered Aggregates on the Mechanical Properties of Ultra-High-Strength Concrete. Materials.

[50] Odler I., Hewlett C.P. (1988). Lea’s Chemistry of Cement and Concrete.

[51] Ju Y., Zhang H., Wang D., Kong X., Ma Y., Zhang X., Bai J. (2024). Effect of Mineral Admixtures on the Resistance to Sulfate Attack of Reactive Powder Concrete. J. Clean. Prod..

[52] Zhongya Z., Xiaoguang J., Wei L. (2019). Long-Term Behaviors of Concrete under Low-Concentration Sulfate Attack Subjected to Natural Variation of Environmental Climate Conditions. Cem. Concr. Res..

