# Supplementary material for: Evaluation of Cement Composites with Heavy Metal-Contaminated Recycled Aggregate: Toward Sustainable Utilization
Source: Materials (Basel). 2025 Dec 9;18(24):5533. doi: 10.3390/ma18245533 (PMC12734575; doi:10.3390/ma18245533)
Supplement: Supplementary file 1 [file materials-18-05533-s001.zip › materials-3991395-supplementary.pdf]

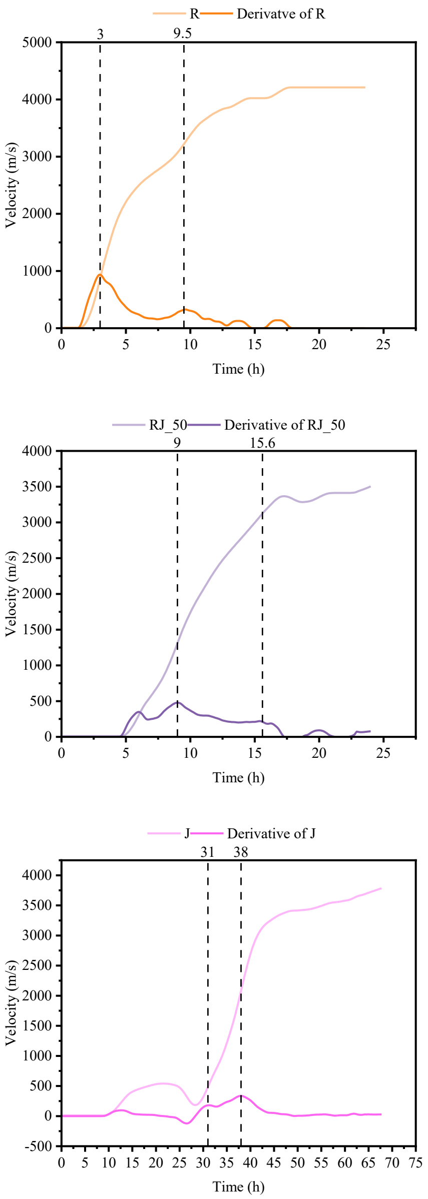

Figure S1. Determining the initial and final setting times using the first derivative of ultrasonic pulse velocity (UPV). The local maxima observed in the first derivative correspond to distinct phases formed during cement hydration.

Sieve size	ZnS (%)	Anglesite (%)	Cerrusite (%)	Descloisite (%)	Epsomite (%)	Fluorite (%)	Galena (%)	Gypsum (%)	Hemimorphite (%)	Ghoetite (%)	Hydrozincite (%)	Marcasite (%)	Melanterite (%)	Pyrite (%)	Smithsonite (%)
4 mm	0.4	0.3	0.5	0	1.4	2.1	0.4	0.8	0.3	0.4	0.5	0.9	2.2	1	0
2 mm	0.5	1.3	0.7	0.1	2.4	2.7	1.9	6.7	0.6	2.8	0.4	0.8	0.1	6.8	0.3
1 mm	0.3	1	0.9	0.1	1.1	2.7	0.9	0	2.9	0.2	6.2	0.1	0	2.8	1.3
500μm	0.9	0.2	0.3	0.4	1.1	1	1	0	0.1	0.1	0.5	2.4	3.7	5.4	0
250 μm	0.9	1.6	0.5	0.2	0.4	0.6	0.2	1.1	3	0.3	0.6	0.1	3.3	1.6	0
125 μm	1.1	0.1	0.4	0.5	6.7	5.4	0.6	0.1	2.5	0.1	5	0.2	0.6	6.5	0.2
63 μm	1.5	0.2	0.5	0.1	0.1	3.1	0.2	0.4	2.1	0.6	1	0	1.2	4	0.1
Residue	1.6	1.2	0.3	0.6	6	3.3	0.2	0.3	1.7	0.9	1.2	0.1	2.6	3.3	0.2

Table S1. Mineralogical composition of the recycled aggregate J determined by XRD.
